# The Newest Federalism and Population Health

**DOI:** 10.1111/1468-0009.70124

**Published:** 2026-08-27

**Authors:** JENNIFER KARAS MONTEZ, JACOB M. GRUMBACH

**Affiliations:** ^1^ Syracuse University; ^2^ University of California ‐ Berkeley

**Keywords:** New Federalism, health policy, population health, federalism, social determinants of health, social policy

## Abstract

**Policy Points**

- In the United States, social policies at the federal, state, and local levels affect population health. The country is undergoing major changes in its social policies and intergovernmental relations.
- As both state and local governments have taken different approaches to social policies, where one resides and the socioeconomic resources one has increasingly have life and death consequences.
- The following three contemporary changes in intergovernmental relations may have profound consequences for population health: erosion of the federal floor, formation of cross‐state alliances, and circumvention of congressional policymaking.

**Context:**

American federalism involves decentralized policymaking across federal, state, and local governments. Since the 1980s, changes in those intergovernmental relations, termed “New Federalism,” combined with polarization in policies across state and local governments, resulted in some places being conducive to health while others are not. This Perspective proposes that the country has entered “Newest Federalism” with contemporary changes in intergovernmental relations that carry profound, and largely unstudied, implications for population health.

**Method:**

Drawing on the federalism, health policy, and population health literatures, this Perspective links New Federalism and state policy polarization to the troubling trends and growing disparities in population health since the 1980s. It then identifies three features of the current context, coined the Newest Federalism, that will adversely impact population health.

**Findings:**

Three characteristics of the Newest Federalism are particularly important. These include the erosion of the federal floor, evident in the declining real minimum wage, repeal of federal abortion protections, cuts to SNAP and Medicaid, threats to the Department of Education, and more; formation of cross‐state alliances that fill federal policy vacuums in areas such as public health, data privacy, and reproductive rights, but that may themselves widen disparities between states; and growing use of executive orders, agency actions, and waivers to circumvent congressional policymaking. These changes may harm population health overall and disproportionately affect people with limited socioeconomic resources and those living in states that cannot or will not offset federal retrenchment.

**Conclusions:**

Understanding and mitigating the health consequences of the Newest Federalism will require research that examines the combined effects of federal, state, and local policy change, attends to the distributive consequences of cross‐state alliances, and informs policymakers and the public about the stakes for population health.

American federalism involves decentralized policymaking across federal, state, and local levels, which produces striking policy variations across geography. Federal policy often creates a so‐called federal floor in a policy area, such as a federal minimum wage, below which states and localities cannot go. From the 1930s through the 1970s, the federal government established and expanded such floors through the implementation of major programs such as Social Security, unemployment insurance, Medicare, and Medicaid.

However, American federalism is not static. After that half‐century of federal action, American federalism underwent significant changes. By the 1980s and 1990s, policy changes at the state level were especially noteworthy, as states “moved from the margins to the center of partisan battles over the direction of US public policy.”^1^ States became major policymakers from that time onward for multiple reasons. Key reasons include the devolution of certain policymaking authorities from the federal government to state governments as part of the “New Federalism” movement; states’ escalated use of preemption laws to block local policymaking authority; and the lobbying of organizations favoring corporations to modify US policy “one state capital at a time.”^1^, ^2^, ^3^

As a continuation of this transformation of federalism, liberal states and cities created new social policies to substitute for the lack of progress in raising the federal floor in domains such as minimum wage and paid sick leave. Whether focused on state‐level implementation of federal policy, as in Medicaid expansion under the Affordable Care Act, or on their own policy innovations, as in new tobacco regulations, these movements helped generate striking differences among states and localities in their social policy contexts.^1^, ^4^

In no area are the consequences of this transformation of American federalism clearer than in population health. Social policies at the US federal, state, and local levels influence population health. They do so by affecting nearly every aspect of people's lives, such as prenatal health care, K–12 education, access to tobacco and firearms, exposure to second‐hand smoke, labor standards such as minimum wage levels and paid leave mandates, economic tax structures, safety nets, abortion access, and vehicle emissions standards. Importantly, some social policies affect the health of some people and places more than others. For example, cigarette taxes affect mortality rates among people with low, but not high, levels of education^5^; earned income tax credits affect birth weights among Black, but not White, women^6^; and state preemption of local authority to mandate paid leave affects mortality in urban, but not rural, areas.^7^ Policy changes such as these can be understood as part of a transformation of American federalism since the 1980s that has affected overall population health as well as disparities in health across people and places.^8^, ^9^

The nation is once again undergoing seismic changes in its policy context and intergovernmental relations. The constellation of changes is unlike those in any historical moment. For one, the erosion of the federal floor appears to be accelerating. Alongside ongoing erosions, such as the multidecade decline in the real value of the federal minimum wage, more recent erosions includes the 2022 repeal of the constitutional right to an abortion, new work requirements for Medicaid beneficiaries enacted under HR 1,^10^ and HR 1's significantly reduced federal funding for the Supplemental Nutrition Assistance Program (SNAP).^10^ A second change is that states have been building cross‐state alliances in domains such as public health, abortion, data privacy, and emissions standards to fill a federal policy vacuum. Third, particularly in recent presidencies, congressional policymaking has been circumvented by executive orders, presidential memoranda, hiring and firing of agency personnel, legal threats and withholding of resources for states that do not comply, plus other tools of an administrative presidency.^11^, ^12^ The combined impact on population health may be profound, especially for certain states and localities and for people with limited socioeconomic resources.

This article has three aims: (1) to draw attention to recent changes in intergovernmental relations and social policy contexts, (2) to spawn research on how those changes impact population health, and (3) to generate awareness on the need to prevent or mitigate adverse health impacts. It begins by highlighting key changes in federal–state (and local, to a lesser extent) intergovernmental relations in recent decades and how they have exacerbated long‐standing differences in states’ social policies. The next section discusses the impact of those changes on population health across geographic contexts and individuals’ socioeconomic status. The last section highlights three contemporary changes that are especially salient for population health: erosion of the federal floor, formation of cross‐state alliances, and circumvention of congressional policymaking.

## New Federalism and State Policy Divergence

Federalism is a system of multiple levels of government, each of which has separate constitutional authority. Distinct from government‐to‐government relationships between separate sovereigns, as in Tribal–federal relations, the US Constitution creates federal and state authority. Although states have no constitutional authority on their own, they further delegate authority to county and local governments, further decentralizing policymaking.

The relative importance of uniform national policy and administration, on one hand, and more varied state and local policy and administration, on the other, has shifted over American history. Programs enacted from the New Deal through the Great Society (1930s through 1960s) made policy contexts more similar across states, although the state‐level administration of New Deal programs such as unemployment insurance and the minimum wage generated racial and regional inequality.^13^ As a reaction, the Nixon and Reagan administrations championed what they called “New Federalism,” placing an emphasis on greater state‐level discretion, such as in the provision of federal grants as block grants instead of categorical grants. Compared with categorical grants, which tend to have strict program rules and federal oversight, block grants give states flexibility on program design and administration. Importantly, the design of block grants has evolved in fundamental ways over time. The block grants of the 1960s and 1970s tended to provide more funding than the federal grants they replaced, whereas those of the 1980s often contained less funding. Many block grants of the 1990s provided less funding and eliminated individual entitlements.^14^ A prime example is the 1996 Personal Responsibility and Work Opportunity Reconciliation Act. It replaced Aid to Families with Dependent Children and related safety‐net programs, which were individual entitlements, with Temporary Assistance for Needy Families (TANF), which gave states greater discretion over eligibility and administration and set both time limits on receiving benefits and work requirements to qualify for benefits.

The political discourse around federalism has also changed over time. For much of the post–World War II period, federal devolution of certain authorities to the states was associated with conservative politics, whereas expansions of federal authority were associated with liberal politics. However, this alignment has recently become muddled. Conservative policymakers, as well as both the Biden and Trump administrations, embraced an expansive federal executive to override state and local policies when desired. At the same time, politically liberal state and local governments have embraced devolution to advance liberal‐leaning policies, such as higher minimum wage levels and reproductive health protections, for which there is a federal policy vacuum. This reshuffling means that federalism debates no longer map cleanly onto a single ideological dimension.

The deployment of New Federalism exacerbated long‐standing differences across states in the generosity of their safety‐net programs. An analysis of 10 programs found that the largest differences were in the programs for which states were given greater financial and administrative responsibility.^15^ That analysis highlighted the striking expansion of cross‐state differences in the generosity of programs associated with the 1996 Personal Responsibility and Work Opportunity Reconciliation Act. As an example, in 1980, the Aid to Families with Dependent Children benefit for a three‐person family was five times greater in the most generous state compared to the least generous state, which were Vermont and Mississippi, respectively.^16^ In 2023, the TANF benefit was six times greater in the most generous state compared to the least generous state, which were California and Arkansas, respectively. The divergence is evident in myriad other safety‐net policies. In 1980, no state offered an earned income tax credit (EITC). By 2023, 31 states offered an EITC but with a wide range of generosity levels, ranging from 3% of the federal credit in Montana to 125% of the federal credit in South Carolina.^16^ In 1980, the maximum unemployment benefit per year ranged from $7,759 to $19,596 in 2023 dollars, a 2.5‐fold difference. In 2023, the benefit ranged from $5,719 to $45,660, an 8‐fold difference.^17^

The growing variation in state policies is not limited to social safety‐net policies. States diverged further away from each other than they had been historically in numerous social policies beyond the safety net, such as minimum wage levels, right to work laws, abortion access, tobacco taxes, and firearm safety laws. Such policy decisions are driven by many factors, such as states’ fiscal capacities^18^ and balanced budget requirements,^19^ lobbying from organizations such as the American Legislative Exchange Council,^2^ and states’ political party control.^20^ States and localities also enacted various immigration‐related laws, despite the fact that the only the federal government has the authority to create and enforce immigration laws. For example, some states now allow, whereas others prohibit, driver's licenses and in‐state tuition for undocumented persons, and some localities have designated themselves as sanctuary cities that forbid or limit cooperation with federal immigration officers. The net result of the myriad policy changes is that some state and local governments have created a policy context that invests in individuals’ well‐being, whereas others have not. For instance, some states raised the minimum wage, increased tobacco taxes, enacted robust firearm safety laws, mandated paid leave, expanded Medicaid, and more, whereas many other states did not enact such policies and some decided to prohibit their localities from doing so.^3^ Consequently, the policy context of most states has shifted further to the left or to the right of the political spectrum.^1^, ^9^ In other words, some states (typically Democratic led) have enacted a bundle of policies that promote health, whereas many others (typically Republican led) have not.

## New Federalism and the Social Determinants of Health

One consequence of New Federalism combined with state policy polarization is a heightened salience of certain social determinants of health. This section focuses on two such determinants, namely, geography and socioeconomic status, and their intersection.^21^ Since the 1980s, these factors have become increasingly powerful predictors of population health. As state governments have taken different approaches to their social policies, where one resides and the socioeconomic resources one has increasingly have life and death consequences. Although local governments have also taken different policy approaches, the focus of this discussion is on states.

### Geographic Context

Disparities in population health across geographic areas of the country have fluctuated over time. For example, life expectancy was converging across states during the 1960s and 1970s, but it began diverging in the mid‐1980s.^9^ The difference in life expectancy between the longest‐ and shortest‐lived states grew from 5.9. to 8.5 years between 1960 and 2022.^22^ The convergence–divergence trend in states’ life expectancies mirrors trends in both New Federalism and policy polarization. Regarding New Federalism, as mentioned previously, block grants in the 1960s and 1970s tended to provide more funding to states than the federal grants they replaced, whereas those starting in the 1980s contained less funding and allowed more state discretion.^14^ Regarding polarization, during the 1960s and 1970s, the policy contexts of states were converging on a liberal–conservative orientation, but they then diverged starting in the 1980s.^1^, ^9^

How might New Federalism, combined with state policy polarization, have elevated the importance of geography as a social determinant of health? For one, whether someone is exposed to a policy context that promotes health is increasingly determined by their state of residence.^1^ Table 1 compares key social policies in Massachusetts and Tennessee. In 2023, Massachusetts and Tennessee differed on 11 of the 13 policies. Policies in Massachusetts were more health promoting than those in Tennessee, particularly for low‐income people. A two‐person family in Massachusetts could qualify for a TANF benefit of $692 ($343 in Tennessee). Under Massachusetts law, many workers are covered by mandated paid sick leave (not mandated in Tennessee), eligible low‐ and moderate‐income workers can claim a supplemental EITC (not offered in Tennessee), the minimum wage is $15 per hour ($7.25 in Tennessee), and annual unemployment benefits can reach up to $45,660 ($7,150 in Tennessee). Residents of Massachusetts may receive expanded Medicaid benefits and are discouraged from smoking ($3.51 tax per pack of cigarettes vs. $0.62 in Tennessee) and risky firearm use. Table 1 also shows that contemporary policy differences between Massachusetts and Tennessee did not always exist. In 1980, these two states differed in just four of the 13 policies, and the differences tended to be small.

**TABLE 1:** Key Social Safety Net, Labor/Economic, and Health Behavior Policies in Massachusetts (MA) and Tennessee (TN) in 2023 and 1980

	2023		1980	
Policy	MA	TN	MA	TN
AFDC/TANF monthly benefit for 2‐person family ($)	692	343	1041	322
Child tax credit	yes	no	no	no
Paid sick leave	yes	no	no	no
EITC rate as a percentage of the federal rate (%)	30	0	0	0
Right to work law	no	yes	no	yes
Minimum wage ($)	15.00	7.25	10.28	10.28
Maximum unemployment benefit per year ($)	45,660	7,150	19,596	8,621
Number of six labor‐related preemption laws enacted	1	6	0	0
Affordable Care Act Medicaid expansion	yes	no	no	no
Tobacco tax ($)	3.51	0.62	0.70	0.43
Firearms				
Child access protection law	yes	no	no	no
Stand your ground law	no	yes	no	no
Right to carry law	yes	yes	no	no

AFDC, Aid to Families with Dependent Children; EITC, earned income tax credit; TANF, Temporary Assistance for Needy Families.

*Notes*: All dollar amounts are shown in 2023 US dollars. The Affordable Care Act's Medicaid expansion began on January 1, 2014. All data are from the State Policy & Politics Database,^17^ except for the child tax credit information, which is from the Tax Policy Center.^23^

As demonstrated by this example, the state in which one resides has become an important social determinant of health, especially for low‐income individuals and families. State policies such as those listed in Table 1 can affect health directly and indirectly. For example, not only does Medicaid expansion improve access to health care, it also reduces the likelihood that low‐income adults must choose between medical care and other necessities, and it reduces the likelihood of evictions and unpaid bills.^24^ Access to paid sick leave improves health directly by lowering the odds of forgoing needed medical care^25^ and indirectly by lowering the odds of job loss and economic hardship.^26^ Receipt of an EITC helps offset the costs of essentials such as housing and food, reduces debt, reduces smoking, and improves high‐density lipoprotein (“good”) cholesterol levels and immune function, among other benefits.^27^, ^28^ These are a few examples of the many social policies that make Massachusetts a fundamentally differently policy context than Tennessee, a difference experienced most acutely by low‐income individuals and families.

Such differences in states’ policy contexts appear to have affected both population health overall and geographic disparities in health. One analysis of changes in 17 policy domains (e.g., labor, civil rights) during the period from 1970 to 2014 concluded that, in some years, the changes were net positive for US life expectancy, but in most years, they were not.^9^ This analysis estimated that the trend in life expectancy after 2010 would have been 25% steeper for women and 13% steeper for men if states’ policies had not changed in the way that they did. Policies on tobacco, labor, immigration, civil rights, abortion, and the environment were particularly important predictors of life expectancy. Changes in states’ social policies also appear to have contributed to widening differences in population health between geographic areas. Examining explanations for the widening disparities among states in working‐age mortality rates, Couillard and colleagues concluded that “the most promising explanation [for the widening disparities] involve[s] efforts by high‐income states to adopt specific health‐improving policies and behaviors since the 1990s.”^8^ Changes in states’ social policies also appear to have widened disparities in working‐age mortality rates between counties. The divergence between states in their firearm laws, tobacco taxes, EITC generosity, and right‐to‐work laws also contributed to the widening disparities between counties from 1990 to 2019.^29^

Although the focus of this article is on states, it is important to note that policies have also changed at the local level in many places. As a few examples, some cities and counties have enacted sanctuary policies to protect immigrants, some have raised their minimum wages above the state level, and some have enacted more stringent firearm safety laws. Tribal areas have also been innovating on policies that impact health, such as the Navajo Nation's tax on junk food enacted in 2014. In sum, the chances of living a long and healthy life are increasingly tied to one's place of residence.

### Socioeconomic Status and Its Intersection with Geography

Since the 1980s, population health has also become more disparate across socioeconomic status. Using educational attainment as a marker of socioeconomic status, studies have documented the growing divide in health across education. One study estimated the number of years that adults could expect to live between ages 25 and 75 years for different education levels.^30^ This study concluded that, in 1990, adults with at least a bachelor's degree could expect to live 1.9 years longer than those without a degree. By 2018, the difference had grown to 3.1 years. This difference grew because life expectancy rose for those with at least a bachelor's degree but declined for those without a degree.^30^

How might New Federalism, combined with state policy polarization, have elevated the importance of socioeconomic status for health? Quite simply, the retrenchment of federal funding for safety‐net programs and the devolution of those programs to the states primarily impacted low‐income individuals. Means‐tested safety‐net programs, such as TANF and SNAP, are, by definition, relevant only for low‐income individuals. Because low income correlates strongly with low educational attainment, these programs are also disproportionately relevant for less‐educated individuals.^31^ Retrenchment affects people's chances of experiencing poverty and of alleviating poverty's deleterious effects through access to food, health care, and other essentials.

Social policies are powerful levers for modifying the importance of education for health. Policies can reinforce or disrupt the pathways through which education affects health, namely, economic conditions, health behaviors, psychosocial factors, and health care. For example, policies might disrupt the pathway through economic conditions by increasing the minimum wage and offering an EITC. They might disrupt the pathway by means of behaviors by raising tobacco taxes, increasing SNAP benefits, and bolstering firearm safety laws. They might also disrupt the pathway by means of psychosocial resources through paid leave mandates, eviction protections, and access to reproductive health care. In these examples, policies reduce the adverse effects of low education on health.

The facts that social policies are increasingly different across states and that these differences are primarily salient for less‐educated persons are evident in the patterns of health disparities. For example, the association between education and health is much stronger in some states than others.^32^, ^33^, ^34^ It tends to be strongest in states with relatively weak labor protections and safety nets. Moreover, the health of *less‐educated* adults is more disparate across states than is the health of *more‐educated* adults.^32^, ^33^, ^34^ In addition, the association has grown the most in states where higher education has become increasingly necessary for employment and economic well‐being.^35^ Social policies in states such as Massachusetts help disrupt the pathways between education and health, whereas policies in states such as Tennessee strengthen them.

The retrenchment and devolution of safety‐net programs can disproportionately affect low‐income, low‐educated individuals for additional reasons. One of these reasons is a higher tax burden. When financial responsibility for safety‐net programs shifts to the states, one strategy that some states use to cover the cost is to raise taxes. Because state taxes are generally regressive compared to federal taxes, especially in conservative states, low‐ to moderate‐income individuals may pay a larger share of their income for such programs when funding and responsibility shift to the states.^36^ Regressive state taxes, in turn, predict higher mortality rates among poor individuals as well as higher rates of crime, obesity, teen pregnancy, and high school dropouts.^37^ In sum, shifting responsibility for safety‐net programs to the states has had negative outcomes for low‐income individuals across many states. As Gillman stated, “power to the states is usually disempowering for the poor.”^38^ ^(p85)^

Thus, New Federalism combined with policy polarization sparked a divergence in states’ policy contexts. Consequently, the state in which one resides and the education one achieves are increasingly strong predictors of how long and how healthily one will live. People residing in increasingly liberal state policy contexts and those with at least a bachelor's degree have generally fared best and experienced improvements in many markers of health. However, federalism is changing again in key ways. Population health researchers have yet to define what these changes mean for the future of US health and social disparities in health. The next section discusses these changes.

## The Newest Federalism and the Future of Population Health

The push for greater state policy discretion has continued into the modern era. By George W. Bush's first term, David Plotz called Bush's version “The New, New, New, New, Federalism.”^39^ This section uses the term “newest federalism” to refer to contemporary changes in intergovernmental relations and the policy environment. The changes will likely impact the health of the population overall. They may also exacerbate disparities in health across people by disproportionately affecting less‐educated people and those residing in certain US states. This section discusses three key characteristics of the contemporary context that portend trouble for population health.

### Erosion of the Federal Floor

The term “federal floor” is often used to refer to minimum regulatory protections set by the federal government or Supreme Court, such as for same‐sex marriage and abortion access,^40^ and to minimum resource standards, such as the federal minimum wage.^41^ It is also used to describe the intersection between federal and state laws, as the former sets the floor.^42^, ^43^ This section highlights some ways in which the floor has eroded in both protections and resource standards.

The federal government has not raised the minimum wage since 2009, now the longest stretch without a raise since the Fair Labor Standards Act was enacted in 1938.^44^ Today's value of the wage ($7.25 per hour) is about half of what it was in the 1960s; in 1968, the wage was $14.80 in 2025 dollars. Lacking federal action, many states have raised their minimum wage. In 2026, it ranges from $17.13 in Washington state to $7.25 in states with a minimum wage set at or below the federal level or no minimum wage at all. Even though some states raised their minimum wage above the federal level, the real value of the wage has nevertheless eroded below its 1968 level in 33 states. In other words, the floor has fallen for minimum‐wage workers across most states. Former President Biden's efforts to raise the federal minimum wage to $15 were unsuccessful. In a 2021 executive order, he raised the minimum wage for federal contractors; however, this effort was later undone by Trump in another executive order.^45^

Additional erosion of the federal floor occurred in 2022 when the Supreme Court repealed the constitutional right to an abortion. As of January 6, 2026, the status was 13 states with bans on abortion, six states with gestational limits of 6 to 12 weeks, and four states with gestational limits of 18 to 22 weeks.^46^ That is, in about half of states, the federal floor protecting the right to an abortion has been eliminated or severely damaged. The consequences will be disproportionately borne by low‐income women. An analysis of the 2020‐2021 Abortion Patient Survey found that nearly three‐fourths of individuals obtaining abortions had incomes below 200% of the federal poverty level.^47^

Core safety‐net programs are also being eroded. For example, the budget reconciliation act of 2025 (HR 1^10^) targeted SNAP and Medicaid by cutting federal funding, restricting eligibility, expanding work requirements, and more. It cut spending on SNAP through 2034 by 20%, which is the largest cut in SNAP's history, and requires adults to work at least 80 hours per month or be punished with a three‐month time limit for benefits across a three‐year period.^48^ It also hamstrings states—a move more aligned with making the program unsustainable than with allowing states to decide how to best implement the program. For instance, HR 1 removed the “insufficient jobs waiver,” which had long allowed states to provide SNAP benefits beyond the typical three‐month limit for recipients living in areas with few work opportunities or high unemployment rates.^49^ It also shifted a substantial cost burden onto the states, requiring states to now pay the majority of administrative costs, contribute a percentage of the benefits, and pay hefty penalties for errors.^48^

The federal floor provided by the US Department of Education (DOE) is also being undermined. Current efforts by the executive branch to restrict funding and shift operations of the DOE to other federal agencies or the states may have devastating consequences. This is particularly worrisome if efforts to dismantle the DOE are enacted by Congress. Roughly 15% of K–12 students with disabilities could lose their right to a “free appropriate public education,” and public schools with high shares of low‐income students that receive federal assistance (which is more than 50% of public schools) may lose that financial lifeline.^49^, ^50^ Lacking this federal floor, states and localities will need to decide whether to continue these long‐standing protections and, if so, how to fund them. Given the importance of education for people's economic well‐being, labor force participation, health, medical care costs, and more, the impact may be profound for the country as a whole. It will be especially so for low‐income people in states that shrink support for and access to K–12 education.

### Cross‐State Alliances

A second key characteristic of the contemporary context is cross‐state alliances that aim to fill the policy and regulatory vacuums created by the erosion of the federal floor and certain federal agencies. Some cross‐state alliances share resources and information (e.g., public health data, data privacy enforcement), whereas others define shared policies and regulations (e.g., vehicle emissions standards), and some cooperate on legal actions against the federal government (e.g., multistate lawsuits against the federal government). To be clear, cross‐state alliances are not new. However, several such alliances have either launched or expanded and formalized in recent years, in response to the erosion of the federal floor, the dismantling of federal agencies, and the lack of federal regulations.

In 2025, three cross‐state alliances focused on public health launched to coordinate guidelines, share resources, prepare for future emergencies, and more.^51^ They are the West Coast Health Alliance, the Northeast Public Health Collaborative, and the Governors’ Public Health Alliance. The Washington state governor's office announcement on the launch of the West Coast Health Alliance explained that it “represents a unified regional response to the Trump Administration's destruction of the U.S. Centers for Disease Control and Prevention's (CDC) credibility and scientific integrity.”^52^ It has already disseminated evidence‐backed statements endorsing certain vaccine schedules, countering claims that vaccines cause autism, and detailing concerns about recommendations made by the Centers for Disease Control and Prevention's new Advisory Committee on Immunization Practices.^53^

The Consortium of Privacy Regulators was launched in 2025, with 10 member states at present. Mainly consisting of state attorneys general, the consortium aims to build a more robust consumer protection apparatus by sharing expertise and resources, facilitating discussion and debate about privacy law developments, harmonizing data privacy laws across states, and investigating alleged violations of privacy laws among member states.^54^ Its importance is underscored by the lack of a cohesive federal regulation and efforts by the Trump administration to dismantle the Consumer Financial Protection Bureau through mass firings and furloughs.

In 2023, the year after the constitutional right to an abortion was overturned, the Reproductive Freedom Alliance was launched. It currently includes 22 of the 50 states as members. Its objective is to “share best practices, coordinate responses to threats and attacks on reproductive rights, and advance affirmative strategies to protect, strengthen, and expand access to abortion, contraception, and other forms of reproductive health care.”^55^

Such alliances are laudable and function as an important stopgap measure. At the same time, they may have unintended consequences. One potential consequence is an exacerbation of health disparities between states. For example, to date, the three cross‐state public health alliances mentioned previously include just 16 states. All 16 are Democratic‐leaning and tend to have health‐promoting policy contexts and healthier populations.^9^ In other words, the residents of the healthiest states are the ones who will benefit from the alliances. Second, the alliances may exacerbate health disparities between socioeconomic groups within states that are not members of the alliances. As explained previously, states’ policy contexts appear to be particularly critical for the health of less‐educated individuals. Such individuals residing in non‐alliance states may be disproportionately harmed by the erosion of (trusted) federal guidance on public health matters if their state does not step in to fill the void.

### Circumventing Congressional Policymaking

A third characteristic of the current environment that will impact population health is the increasing tendency for presidents to enact their agendas outside the legislative process. Recent presidents have done so by circumventing Congress using tools of an administrative presidency such as executive orders, presidential memoranda, and the hiring and firing of agency personell.^11^ These tools help presidents achieve their goals without the need to change laws or obtain congressional approval.^12^ The rise of the administrative presidency is altering the balance of power between the federal and state governments, expanding the power of the former in multiple domains.^11^

Bypassing legislative processes to enact an agenda can be beneficial if the issue is urgent and has overwhelming public support but congress is impotent. However, there are real downsides. For one, policies imposed by executive order are often overturned by the next administration and are therefore fleeting and disruptive.^11^ In addition, the circumvention strains relations between the federal and state governments and can generate a backlash from states that refuse to implement the policy and/or sue the federal government using their limited coffers.^11^ Bypassing the legislative process also raises the risk that the policy harms population health, as it did not undergo a transparent and robust debate among congress, policy experts, the media, and the public.

Another popular administrative tool used in recent decades for enacting an agenda while circumventing the legislative process is waivers. Waivers, as part of Section 1115 of the Social Security Act, allow federal and state governments to experiment with safety‐net programs such as TANF, SNAP, and Medicaid without changing the underlying federal statutes.^56^, ^57^ Waivers can be used to expand or contract such programs. For example, the Obama administration encouraged state waivers to expand Medicaid, whereas the Trump administration has encouraged state waivers to tighten eligibility and cut coverage.^56^ Most recently, the US Department of Agriculture has been encouraging states to apply for SNAP Food Restriction Waivers. Waivers from 18 states have so far been approved to restrict eligible food items.^58^ Waivers represent an additional strategy for rolling back SNAP, over and above the changes in the 2025 budget reconciliation act.

The increasing use of waivers to curtail safety‐net programs is “accelerating the fragmentation of America's welfare state” and will likely impact population health.^56^ Any impact will, by definition, fall hardest on low‐income populations who need access to such programs to avoid poverty, leave poverty, and mitigate the deleterious consequences of poverty. The impact will also fall hardest on people residing in certain states. These include states that chose to constrict the programs even further through waivers as well as states that may not wish to constrict the programs but do not have the financial capacity to sustain them at their preferred level given federal cuts to funding.

## Implications and Conclusion

The ideas inherent in New Federalism combined with policy polarization have thus generated striking changes in states’ policy contexts, changes that appear to have affected population health overall and social disparities in health. Consequences of the newest federalism may be even more profound. This article identified three characteristics of the newest federalism that may have far‐reaching consequences: erosion of the federal floor, cross‐state alliances, and circumvention of congressional policymaking. All three will disproportionately impact people with limited socioeconomic resources. They will also disproportionately impact people residing in states that do not or cannot compensate for the erosion of the federal floor, do not engage in cross‐state alliances to protect population well‐being, and pursue waivers to further weaken the safety net. Departing somewhat from past versions of New Federalism, the newest version will be felt by all people and places to some degree. For instance, the erosion of the federal floor across domains such as K–12 education and the National Institutes of Health will impact the population at large. Likewise, a reliance on alliances to fill a federal vacuum in domains such as public health and data privacy will also impact the population at large.

The major changes in the current policy environment call for a few modifications in scientific research to understand the impact of these changes on population health. First, focusing on the three characteristics of the newest federalism detailed in the preceding section will help direct research toward some of the most pressing among the seemingly infinite and incomprehensible number of policy changes. Second, the combined impact of federal, state, and local policies on population health should be examined. Policies at all levels of government are undergoing seismic changes. Federal, state, and local policies may have synergistic or offsetting effects on population health. It is conceivable that the impact of changing state policy contexts on health may be greater now than in the past, given the erosion of the federal floor. Third, attention should be focused on a potentially new source of disparities arising from cross‐state alliances. Some alliances (e.g., the West Coast Health Alliance) are regional, whereas others (e.g., the Reproductive Freedom Alliance) are geographically dispersed but mostly politically aligned. The extent to which these alliances improve population health among member states and create health disparities between them and nonmember states is an open and important question.

In conclusion, the United States is undergoing seismic changes in its policy context at federal, state, and local levels. The constellation of changes is unlike that of any historical moment. Unprecedented changes may have unprecedented consequences. The changes are poised to harm the health of the population overall but especially for people with limited economic resources and people residing in states with policies that are not conducive for health. This article identified three characteristics of the changes that may have far‐reaching consequences, namely, erosion of the federal floor, formation of cross‐state alliances, and circumvention of congressional policymaking. Their impacts on population health must be studied and communicated to a range of stakeholders.

## Funding/Support

None

## Conflict of Interest Disclosures

The authors declare no conflicts of interest.
